# Application of next-generation sequencing to determine mutations in candidate genes for congenital eye malformations in the Mexican indigenous population

**DOI:** 10.3389/fgene.2025.1710953

**Published:** 2025-12-18

**Authors:** Iván Antonio García-Montalvo, Diana Matías-Pérez, María José Ruiz-Sanginéz, Juan Carlos Zenteno

**Affiliations:** 1 División de Estudios de Posgrado e Investigación, Tecnológico Nacional de México/Instituto Tecnológico de Oaxaca, Oaxaca, Mexico; 2 Deparment of Genetics, Institute of Ophthalmology Conde de Valenciana, Mexico City, Mexico

**Keywords:** congenital eye malformations, NGS, indigenous Mexican population, genetic diversityand mutations, Mexico

## 1 Introduction

Congenital eye malformations, such as anophthalmia, microphthalmia, and coloboma, constitute a heterogeneous group of eye development defects that contribute significantly to childhood blindness worldwide. These conditions range from the total absence of one or both eyes (anophthalmia) to abnormally small eyes (microphthalmia) and specific structural defects such as coloboma, which results from the failure of the optic groove to close during embryogenesis (Goyal et al., 2025). It is estimated that these malformations affect approximately 2 out of every 10,000 newborns, highlighting the need for early and accurate diagnosis for timely management (Stoll et al., 2024).

The etiology of specific diseases or malformations is diverse, ranging from exposure to teratogens and infectious agents to complex multifactorial factors. In those cases caused by single discrete genetic alterations (i.e., single nucleotide variants, intragenic deletions/duplications, large genomic rearrangements) the identification of the disease-causing variant is of great importance for medical management and family counselling. Various genetic techniques can be used to demonstrate a monogenic cause, including direct Sanger sequencing of candidate(s) gene(s) or massive DNA sequencing techniques (also known as next-generation sequencing, NGS) such as gene panel sequencing and exome or genome sequencing which traditionally rely on sequencing of short DNA fragments (∼250 base pairs). Recently, long-read sequencing, which allows analysis of much longer DNA fragments (in the order of kilobases to megabases) has been introduced for genetic diagnosis of human diseases. Although short-read sequencing identifies most genetic variations, long-read sequencing is better at detecting complex structural changes in the genome, such as inversions, deletions, or significant translocations (Logsdon et al., 2020). Other approaches as optical genome mapping uses marking patterns to scan the genome (Dremsek et al., 2021), and microarrays facilitate large-scale analysis of copy-number variations and single-nucleotide polymorphisms (Carter, 2007). Of note, monogenic congenital eye malformations vary significantly in their complexity and genetic basis. Some, such as anophthalmia, microphthalmia, coloboma, and cyclopia, can involve mutations in one of multiple genes and show high genetic heterogeneity, affecting various molecular pathways during eye development. In contrast, disorders such as aniridia and certain congenital cataracts have a more limited and defined genetic basis, facilitating a more accurate molecular diagnosis through genetic testing. Pathogenic mutations can be distributed across several loci, and in most cases, there is no single gene responsible, making etiological interpretation and diagnosis difficult. This genetic diversity reflects the complexity of eye development and the need to use personalized diagnostic approaches for each type of malformation. Traditional DNA sequencing methods, which often focus on a few genes or regions, are insufficient to detect all variants, especially rare or novel ones (Hussain et al., 2025; Szabó et al., 2025).

In this context, next-generation sequencing (NGS) has revolutionized the molecular characterization of these malformations. NGS allows for the simultaneous analysis of hundreds to thousands of genes with high depth and precision, facilitating the comprehensive detection of potentially pathogenic variants, ranging from point mutations to insertions, deletions, and copy number changes. This multidimensional capability makes NGS a fundamental tool for unraveling the genetic basis of congenital eye malformations, particularly in populations with unique genetic structures (Satam et al., 2023; Qin, 2019).

A clear example is the indigenous Mexican population, characterized by marked genetic diversity, the result of its historical evolution, migrations, geographical isolation, and relative inbreeding, which have generated unique genetic profiles (Moreno-Estrada et al., 2014). This genetic variety can not only influence susceptibility to congenital eye malformations (for multifactorial etiology), but also determine specific allele frequencies and the presence of rare or exclusive variants (for monogenic etiology). However, this population has been poorly studied in the ocular genetics field, limiting the knowledge about predominant disease-causing mutations.

The application of NGS in studies targeting the indigenous Mexican population represents a valuable opportunity to overcome these barriers. This technology allows for the detection of mutations previously described in other ethnic groups, as well as novel variants specific to this community, improving molecular diagnosis, genetic counseling, secondary prevention, and the creation of personalized therapies (Miron-Toruno et al., 2025; Hernández-Pedro et al., 2019). Expanding knowledge about ocular genetics in traditionally marginalized communities helps close gaps in public health and promotes equity in access to advanced technologies.

## 2 Genetic profile and diversity of mutations in candidate genes for ocular malformations in the indigenous Mexican population

The genetic profile of Mexican indigenous populations shows profound heterogeneity and a genetic structure that is influenced by historical, geographical, and demographic factors. Recent studies of the complete genome of the various indigenous groups in Mexico show a marked differentiation between northern and central-southern populations, resulting from processes of isolation, genetic drift, or migration patterns dating back thousands of years, even to the beginning of sedentary agriculture in Mesoamerica (García-Ortiz et al., 2021). This genetic diversity involves a large number of genetic variants that are functionally and medically significant, reflecting the demographic complexity and internal genetic substructure of these populations (Moreno-Estrada et al., 2014; Ávila-Arcos et al., 2020; González-Martín et al., 2015). Recent advances derived from the Mexican Biobank have made it possible to catalog genetic diversity with population resolution and relate it to biomedical traits as well as various pathologies, highlighting the importance of considering this substructure for medical and genomic studies in this population (Sohail et al., 2023).

The study of candidate genes linked to congenital eye malformations in the indigenous Mexican population must be placed within this context of particular genetic diversity, to understand the specific molecular pathways and mutational variants that may contribute to the development of these pathologies, derived from the adaptive evolution of these population groups. The application of NGS techniques has facilitated the detection of pathogenic mutations in candidate genes such as *SOX2*, *CHX10*, *GDF6*, *RAX*, *OTX2*, and *FOXE3*, among others, which are associated with a broad spectrum of eye abnormalities, such as anophthalmia, microphthalmia, coloboma, and other congenital disabilities. Studies conducted in indigenous and mestizo Mexican groups have revealed that some of these mutational variants are recurrent and specific to the local population, broadening the understanding of the role of these genes in ocular morphogenesis.

For example, the recurrent *SOX2* c.70del20 mutation (c.70_89del, p. Asn24Argfs*65), the most common single gene variant causing eye malformations in humans, was first described in Mexican patients (Zenteno et al., 2005; Zenteno et al., 2006).

In another study, 50 Mexican mestizo individuals with a variety of congenital ocular defects were screened for causative variants in the *CHX10*, *GDF6*, *RAX*, *SOX2* and *OTX2* genes. A disease-causing variant prevalence of 16% was identified, including four *GDF6* mutations (one novel), two novel *RAX* mutations, one novel *OTX2* mutation and one *SOX2* mutation. Of note, anophthalmia and nanophthalmia, not previously associated with *GDF6* mutations, were observed in two subjects carrying defects in this gene, expanding the spectrum of *GDF6*-linked ocular anomalies (Gonzalez-Rodriguez et al., 2010).

Similarly, genetic screening in Mexican patients with anophthalmia/microphthalmia has allowed the expansion of the phenotypic spectrum resulting from mutations in major ocular genes, as observed in subjects carrying *SOX2* deleterious variants and dental anomalies (Chacon-Camacho et al., 2015).

In a case-control study in Mexican subjects with microphthalmia/anophthalmia/coloboma (MAC) spectrum the *FOXE3* p. Val201Met variant was shown to be present in 5 out of 104 *FOXE3* alleles from MAC patients (4.8%) and in 3 out of 210 control *FOXE3* alleles (1.42%). Statistical analysis indicated that this particular *FOXE3* variant was associated with congenital eye defects with an OR of 3.5 (CI 0.8–14.9; p = 0.15) (Garcia-Montalvo et al., 2014). Notably, this association reached statistical significance (OR: 3.3 CI: 1.2–8.5; p = 0.02) when combining such results with those obtained by Reis et al. in a previous study which identified the p. Val201Met variant in 4 out of 232 (1.7%) alleles from Asiatic and Hispanic MAC patients and in 5 out of 748 (0.6%) ethnically-matched control alleles (Reis et al., 2010).

In specific communities, founder mutations have been identified in candidate genes that cause congenital eye malformations, notably the p. Y98H mutation in the *FOXE3* gene, which is associated with a high prevalence of sclerocornea, aphakia, and microphthalmia in an isolated indigenous population in central Mexico. This mutation originated approximately 100–130 years ago, and its high frequency is partly explained by patterns of local inbreeding (Pantoja-Melendez et al., 2013).

More recently, application of NGS to a group of 14 Mexican patients (7 familial and 7 sporadic cases) with congenital ocular malformations allowed the recognition of causal variants in well-known microphthalmia/anophthalmia genes (*OTX2*, *VSX2*, *MFRP*, *VSX1*) or in genes associated with syndromes that include ocular defects (*CHD7*, *COL4A1*). It should be noted that a pathogenic variant in *PIEZO2* modifies a transmembrane domain of that protein. This mutation was identified in a Mexican patient with microphthalmia, sclerocornea, and a flat cornea. The presence of this variant in her affected mother confirmed dominant familial transmission. The variant was not found in more than 690 alleles analyzed from healthy Mexicans, supporting that this mutation is related to the disease. Previously, no association between *PIEZO2* and isolated eye defects had been described. The clinical and genetic results of the study, together with predictions indicating a deleterious effect on the protein, support its classification as pathogenic. This finding broadens the spectrum of *PIEZO2*-related alterations and demonstrates the quality of the analysis performed in Mexican patients with congenital eye malformations (Matías-Pérez et al., 2018).

## 3 Clinical and social applications of NGS implementation in genetic diagnosis for eye malformations

Thanks to the application of NGS, it is possible to perform early and accurate genetic diagnoses that allow for the detailed identification of pathogenic variants involved in the molecular etiopathogenesis of these hereditary ophthalmological diseases. This approach facilitates the implementation of clinical strategies based on precision medicine, optimizing therapeutic decision-making in the early stages of the disease. Timely detection is critical, given that these disorders are associated with severe visual deficits and, in many cases, progress to irreversible vision loss if not adequately treated (Popova and Carabetta, 2025; Consugar et al., 2015).

Given the high genetic heterogeneity of these pathologies and the genetic uniqueness of the indigenous population, technologies capable of analyzing multiple genes and detecting rare or specific variants are indispensable. This increases diagnostic capacity and paves the way for early interventions, personalized management, and effective genetic counseling, essential tools for reducing the burden of eye disease. NGS optimizes efficiency in the healthcare system by avoiding repeated and inconclusive tests, focusing resources on precise preventive and therapeutic strategies. Identifying the exact genetic profile allows specialists to guide clinical follow-up, offer targeted treatments, and assess risks in immediate family members (Qin, 2019; Molla and Bitew, 2024).

The inclusion of NGS in programs targeting indigenous communities would represent a significant social advance, as it could overcome historical barriers that have limited access to advanced technologies, thereby contributing to promoting equity and justice in the field of health. However, in the Mexican health system, the use of NGS remains limited and heterogeneous, concentrated mainly in specialized research institutions, highly specialized hospitals, and private laboratories, reflecting the need to expand its access and application in vulnerable populations to reduce health inequalities. A key aspect is respect for the culture and ethical perspective of the communities (Halmai et al., 2025; Stanley et al., 2020). Clear communication of objectives, genuine informed consent, and collaborative work with leaders and representatives ensure trust and acceptance, guaranteeing that the benefits translate into tangible improvements for the community.

The creation of interdisciplinary networks that bring together ophthalmologists, geneticists, bioinformaticians, and social workers promotes a comprehensive approach, not only from a molecular diagnosis perspective but also with psychosocial support and continuous clinical follow-up to support families throughout the process. This integration also opens the door to future research that expands knowledge about new genes and variants, driving the development of innovative and specific therapies, and strengthening the healthcare system’s ability to anticipate and manage these conditions in a contextualized manner.

## 4 Conclusion

In conclusion, congenital eye malformations represent a significant cause of childhood blindness, with a complex and heterogeneous genetic etiology that requires advanced diagnostic tools for better understanding and management. Next-generation sequencing (NGS), particularly whole-exome sequencing, stands out as a key technology that enables simultaneous, in-depth detection of mutations across multiple candidate genes, overcoming the limitations of traditional methods and enabling the identification of rare or unique variants.

This capability is particularly valuable in the indigenous Mexican population, whose remarkable genetic diversity creates unique profiles, with variants that are not found in studies conducted on mestizo or European populations. Detailed knowledge of the genetic profile and mutational diversity in these communities is essential to improve diagnostic accuracy and avoid misinterpretations. The creation and consolidation of specific genomic databases facilitates the differentiation between benign polymorphisms and pathogenic variants, enriching the understanding of eye development and the genetic mechanisms involved. The clinical and social implementation of NGS represents a step forward in reducing health inequalities and promoting inclusion, offering accurate and timely diagnoses, effective genetic counseling, and individualized and culturally sensitive therapeutic strategies. NGS opens new horizons for research and the development of innovative therapies, promoting more personalized, equitable, and inclusive medicine that responds to the real needs of historically underrepresented communities, helping to close gaps in visual and genetic health in Mexico. However, it should be noted that implementing NGS in Mexican indigenous populations faces key practical and ethical challenges, including infrastructure limitations, the need for culturally appropriate informed consent, and the high cost of the technology. These barriers must be considered a core part of Mexico’s public health policies to guarantee fair and respectful access while minimizing the socio-cultural problems in our communities.
